# Measurement equivalence of child feeding and eating measures across gender, ethnicity, and household food security

**DOI:** 10.1186/s40608-018-0192-6

**Published:** 2018-06-11

**Authors:** Marisol Perez, Tara K. Ohrt, Amanda B. Bruening, Aaron B. Taylor, Jeffrey Liew, Ashley M. W. Kroon Van Diest, Tatianna Ungredda

**Affiliations:** 1 Department of Psychology, Arizona State University, 950 S McAllister Avenue, Tempe, AZ 85287-1104 USA; 2 Department of Educational Psychology, Texas A&M University, College Station, TX 77843-4225 USA; 3 Nationwide Children’s Hospital Department of Pediatric Psychology and Neuropsychology, The Ohio State University Department of Pediatrics, Cleveland, OH 44195 USA; 4 Department of Psychology, Texas A&M University, College Station, TX 77843-4235 USA

**Keywords:** Child feeding questionnaire, Child eating behaviour questionnaire, Child feeding, Eating behaviors, Latino, Hispanic, Food security, Gender, Measurement invariance

## Abstract

**Background:**

Although there have been extensive studies that make group comparisons on child eating and feeding practices, few studies have examined measurement equivalence to ensure that measures used to make such group comparisons are equivalent across important group characteristics related to childhood obesity.

**Methods:**

Using a sample of 243 caregivers with children between the ages of 4 to 6 years, we conducted a measurement equivalence analysis across gender, ethnicity (Latino versus non-Latino White), and household food security. The subscales of the Child Feeding Questionnaire (CFQ) and the Child Eating Behaviour Questionnaire (CEBQ) were examined separately using a one factor multi-group confirmatory factor analysis.

**Results:**

For the CFQ, Concern about Child Weight and Parental Responsibility subscales were consistent across all groups examined. In contrast, Pressure to Eat, Restriction, and Perceived Parent Weight subscales varied or fit poorly across the groups. For the CEBQ, Emotional Overeating, Enjoyment of Food, and Satiety Responsiveness performed consistently across the groups. On the other hand, Food Fussiness, Desire to Drink, Slowness in Eating, and Emotional Undereating subscales varied or fit poorly across the groups.

**Conclusions:**

Findings from this study suggest both of these measures need continued psychometric work, and group comparisons using some subscales should be interpreted cautiously. Some subscales such as Food Responsiveness and Parental Restriction may be assessing behaviors that occur in food secure households and are less applicable to food insecure environments.

## Background

Research has demonstrated that contextual factors such as gender, food security (e.g., limited or uncertain availability of nutritionally appropriate foods), and ethnicity play a role in the development of pediatric obesity [1–3]. For example, research has found that when compared to boys, girls have more fat mass with a different fat distribution pattern, are less sensitive to insulin across childhood, and are more susceptible to family and environmental risk factors that contribute to pediatric obesity [4]. Boys, in turn, are more physically active throughout childhood and adolescence, receive more benefits from physical activity, and tend to have lower leptin levels when compared to girls [4]. This suggests there may be differential risk factors and susceptibility across groups such as sex. Research on ethnicity has demonstrated that Latino children and adolescents have higher rates of overweight and pediatric obesity than their non-Latino White counterparts [3]. Similarly, there is an association with food insecurity and higher rates of overweight and obesity in children [2, 5–7]. Despite studies demonstrating the influence of these contextual factors on pediatric obesity, limited research has been conducted to ensure that questionnaires on key risk factors for pediatric obesity are invariant across gender, ethnicity, and food security. Measurement invariance is important, because construct validity is threatened when items of a scale function inconsistently across groups.

Existing cross-sectional and longitudinal research suggests that parental beliefs and feeding practices contribute to pediatric obesity [8–12]. Two commonly used measures to assess parental beliefs and feeding practices are the Child Feeding Questionnaire [9] and Child Eating Behaviour Questionnaire [13]. Both questionnaires have been used to make group comparisons despite limited psychometric research examining the appropriateness of these measures across key contextual factors related to pediatric obesity.

The Child Feeding Questionnaire (CFQ), one of the most widely used scales in the child feeding literatures, assesses parent’s concern about a child’s weight, responsibility for feeding a child, and the extent a parent pressures a child to eat or restricts a child’s food intake [9]. The CFQ was initially developed with a 7-factor model and validated among an ethnically diverse sample of mothers and fathers with children ranging from 2- to 11-years of age [9]. However, a replication study of low-income Latino and African American families with boys and girls failed to replicate the original factor structure and proposed an alternate model [14]. This same study also found cross-cultural conceptual problems resulting in the authors dropping the perceived weight subscales, as well as a number of items in each of the remaining subscales in order to achieve cross-cultural equivalence [14]. Despite these issues, the CFQ has been used to make group comparisons across a number of different groups, such as parents with boys versus girls [15], Latino versus European Americans [11, 16], food secure and food insecure households [5, 17] and among low-income families without an assessment of food security [18, 19].

The Children’s Eating Behavior Questionnaire (CEBQ) is a parent-report questionnaire that assesses individual eating styles of children that have been found to relate to pediatric obesity [12, 13]. The CEBQ was initially developed with an 8 factor model and validated among mothers and fathers with children between the ages of 3- to 8-years old in the United Kingdom [13]. Additional studies have validated the original factor structure among children, with only a slight variation where food responsiveness and emotional overeating at times load onto the same factor [20, 21]. Within the United States, one study replicated the original factor structure among low income families with pre-school aged children and found measurement equivalence across White and Black participants [22]. Yet, another study of low-income Hispanic and African American families failed to replicate the original factor structure [23]. An additional study of minority low-income families has suggested there may be conceptual issues with some of the scales in the CEBQ [24]. Despite these concerns, the CEBQ has been used to make group comparisons across gender [21, 25], and ethnicity [22, 23, 25]. In addition, the CEBQ has been used across socioeconomic status [25, 26]. If there are problems with measurement invariance, validity of the inferences and interpretations of the results associated with the measure may be threatened.

Research has suggested there may be cross-cultural conceptual problems with the CFQ and CEBQ, and highlighted the need to examine measurement equivalence of these widely used scales, particularly among low-income minority groups [24]. The goal of the current study was to examine measurement equivalence of the CFQ and CEBQ across key contextual factors that influence pediatric obesity (gender, ethnicity, food security). To facilitate across study comparisons of both measures, the current study targeted caregivers with children between the ages of 4- to 6-years old. Both measures have psychometric studies that recruited families from preschool centers, thereby increasing our ability to compare our findings to the extant literature [14, 22–24, 27].

## Method

### Participants and procedures

This study includes 243 caregivers (169 maternal caregivers) with children between the ages of 4- to 6-years old who resided in the home with the children the majority of the time. Table 1 displays the sample characteristics. Approximately 51% of the children were male, and 33.6% were Latino. The majority of caregivers (51.5%) reported a monthly household income of $3000 or below. There were 72 children whose caregiver reported household food insecurity. The number of persons per household ranged from 2 to 10 (*M* = 4.27, *SD* = 1.14). Child body mass index (BMI) was calculated as BMI-for-age (age- and sex-specific) using experimenter-measured child weight and height with Centers for Disease Control and Prevention (CDC) growth charts [28]. Among the children, 66.7% had a BMI percentile score below the 85th percentile and considered of healthy weight, while 23.8% of the sample had a BMI percentile score between 85th and 95th and considered overweight, and 9.5% of the children were considered obese with a 95th or greater BMI percentile score.

**Table 1:** Means, standard deviation, and sample characteristics

Characteristic/Scale	Total (*N* = 243)	Males (*n* = 125)	Females (*n* = 116)	Latino (*n* = 81)	Non-Latino (*n* = 160)	Insecure (*n* = 72)	Secure (*n* = 167)
Age	4.80(0.85)						
Income							
0 - $2000	36.4%						
$2001-$3000	15.1%						
$3001-$5000	15.5%						
$5001-$7000	9.2%						
$7001 – more	23.8%						
Child BMI							
Healthy	66.7%						
Overweight	23.8%						
Obese	9.5%						
Caregiver BMI							
Healthy	36.8%						
Overweight	27.1%						
Obese	36.1%						
Child feeding questionnaire							
Perceived Parent Weight	12.81(1.83)	12.75(1.64)	12.86(2.02)	12.85(1.70)	12.78(1.90)	13.29(1.81)	12.60(1.82)
Concern Child Weight	6.67(3.86)	6.53(3.86)	6.83(3.88)	7.59(4.14)	6.21(3.64)	6.97(3.99)	6.53(3.83)
Parental Responsibility	13.26(2.18)	13.12(2.36)	13.41(1.96)	13.51(2.65)	13.14(1.90)	13.71(1.96)	13.06(2.25)
Restriction	27.28(7.82)	27.59(7.91)	26.94(7.73)	27.09(9.05)	27.37(7.14)	28.18(7.99)	26.85(7.77)
Pressure to Eat	10.52(4.44)	10.99(4.47)	10.04(4.38)	10.86(4.14)	10.35(4.59)	11.33(4.72)	10.21(4.30)
Child eating behaviour questionnaire							
Food Responsiveness	11.56(4.03)	11.64(4.07)	11.47(4.00)	11.38(4.87)	11.64(3.54)	12.17(4.96)	11.24(3.44)
Emotional Overeating	6.83(2.93)	6.78(2.87)	6.89(2.99)	6.95(3.41)	6.78(2.66)	7.24(3.44)	6.67(2.68)
Enjoyment of Food	14.09(2.98)	14.07(2.99)	14.11(2.97)	14.09(3.05)	14.09(2.95)	14.21(3.28)	14.01(2.83)
Desire to Drink	9.83(3.21)	10.31(3.19)	9.30(3.15)	10.26(3.29)	9.61(3.15)	10.69(3.32)	9.39(3.05)
Satiety Responsive	15.29(3.29)	15.19(3.33)	15.39(3.28)	14.69(3.59)	15.59(3.10)	14.88(3.52)	15.46(3.14)
Slowness in Eating	11.75(3.12)	11.68(2.93)	11.82(3.32)	11.36(3.46)	11.94(2.93)	11.61(3.20)	11.82(3.05)
Emotional Undereat	10.79(3.21)	10.70(3.18)	10.88(3.24)	10.39(3.13)	10.99(3.24)	10.50(3.34)	10.88(3.15)
Food Fussiness	14.64(5.34)	18.20(5.30)	17.04(5.33)	16.79(4.86)	18.08(5.53)	17.69(5.33)	17.70(5.33)

*Note:* Total = total sample; Insecure = food insecurity; Secure = food security; Income is monthly household income. Child BMI percentile score below the 85th percentile is healthy weight, percentile score between 85th -95th is considered overweight, and 95th percentile score or greater is considered obese. Caregiver BMI was calculated using experimenter-measured weight and height. Data reported as Means (SD)

Using flyers, participants were recruited from waiting rooms of pediatricians’ offices, daycare centers, preschools, and local stores or businesses that were frequented by families. Families called if they were interested in participating in the study and were screened by phone. Caregivers were excluded if (1) they were unable to use English fluently, (2) had a significant disability that would prevent them from completing the tasks in this proposal, such as blindness, or (3) did not have a child between the ages of 4- to 6-years old. Parents completed online questionnaires on their behavior patterns and those of their children, and were paid for their participation.

### Measures

#### Child Feeding Questionnaire (CFQ)

The CFQ is a 28 item measure given to parents that assesses parent’s perceived responsibility for feeding, perceived parent weight across development, concern about child weight and risk for being overweight, food restriction, and pressure to eat [12, 13]. Items are rated on a scale from 1 to 5. Scores on the subscales range from 4 to 20 for Perceived Parent Weight, 3 to 15 for Concern about Child Weight, 3 to 15 for Parental Responsibility, 8 to 40 for Restriction, and 4 to 20 for Pressure to Eat. Confirmatory factor analyses have tested the factor structure of this measure across Caucasian and Latino samples [14]. Among Caucasian and Latino samples, internal reliability coefficients range from .70 to .92 [14]. In the current study, internal reliability coefficients are: .64 Parent Perceived Weight, .85 Concern about Child weight, .88 Parental Responsibility, .81 Restriction, and .75 Pressure to Eat.

#### Children’s Eating Behaviour Questionnaire (CEBQ)

The Children’s Eating Behaviour Questionnaire is a 35 item measure that assesses parents’ perceptions on child’s eating behaviors with items rated on a scale from 1 to 5. The subscales include child’s responsiveness to food (scores range 5–25), enjoyment of food (scores range 4–20), satiety responsiveness (scores range 5–25), slowness in eating (scores range 4–20), food fussiness (scores range 6–30), emotional overeating (scores range 4–20), emotional undereating (scores range 4–20), and desire for drinks (scores range 3–15) [13]. Past research reports internal reliability coefficients ranging from .74 to .91 [13]. In the current study, internal reliability coefficients are: .79 Food Responsiveness, .86 Emotional Overeating, .82 Enjoyment of Food, .87 Desire to Drink, .71 Satiety Responsiveness, .74 Slowness in Eating, .73 Emotional Undereating, and .88 Food Fussiness.

#### Food security

The United States Department of Agriculture Household Food Security questionnaire was used to assess food security [29, 30]. This is an 18 item questionnaire that categorizes families into high food security (score of 0), marginal food (scores of 1–2) security, low food security (scores 3–7) and very low food security (scores of 8 or more). This measure has been used with different ethnic groups [31]. For this study, individuals with scores of 2 or less were considered food secure, and individuals with a score of 3 or more were considered food insecure. However, among those with a score of 1 or 2, if caregivers reported skipping meals or not eating so that their children may eat, they were classified as food insecure.

### Data analytic approach

Descriptive analyses were conducted using SPSS software. Descriptive statistics were calculated by gender, ethnicity, and household food security via independent sample *t* tests. Internal consistency was calculated and reported across all of the groups.

#### Power analyses

Research has determined that no general rule of thumb will suffice when determining the needed sample size for CFA [32, 33]. Research has found that communality of indicators (i.e., reliability of the indicators), and factor overdetermination (i.e., number of factors/number of indicators) are important when determining sample size requirements for CFAs [33, 34]. MacCallum et al. suggested communalities of .6 or greater, and a minimum of 3 indicators per factor [34]. Using Monte Carlo data simulation techniques, Wolf and colleagues [32] found that for CFAs with one factor loading of .50 and with 3 or 4 indicators required a sample size of 190 or a sample size of 90 if the indicators increased to 6 or 8. Given the findings from Wolf and colleagues [32], our sample size should be sufficient. To further ensure sufficient power, retrospectively the RMSEA analyses were all entered into the Preacher and Coffman online software and yielded power of .80 through .98 [35].

#### Measurement invariance

To conduct the measurement equivalence analyses, confirmatory factor analyses (CFAs) in Mplus [36] were conducted following the procedures recommended by Mulaik and Millsap [37]. For all analyses, we used full information maximum likelihood to handle missing data as this method produces more unbiased results [38]. Little MCAR tests with expectation-maximization methods were performed to evaluate if data was missing at random. For the CFQ, missing data for all the items ranged from 1.2 to 3.2%, and analyses indicate the data is indeed missing at random, χ^2^ (247) = 225.451, *p* = .83. For the CEBQ, missing data for all items ranges from 1.6 to 2.8% and analyses indicate the data is missing at random, χ^2^ (101) = 116.648, *p* = .14. We evaluated model fit using various fit statistics, including the chi-square significance test [39], the root-mean-square error of approximation (RMSEA) [40], and the comparative fit index (CFI) [41]. The Akaike Information Criterion (AIC) was used to compare different models with the lowest AIC value relative to another model is the optimal model [41]. Adequate fit was considered to be a lack of significance on chi-square difference test, a RMSEA <.08, and CFI > .90 [41, 42]. To examine measurement invariance across groups (males vs. females, Latino vs. non-Latino Whites, food secure vs. food insecure) we used a step-wise approach (instead of constraining all the parameters) to identify at which point invariance is no longer achieved between the two groups [43]. The first step entails examining single group solutions of each subscale of the CFQ and CEBQ for each subgroup. For example, we examined Pressure to Eat subscale of CFQ among males and females, separately. If model fit was adequate for each of these samples, then we proceeded to the next step; otherwise, we stopped. The second step involved examining configural invariance, which assesses if the number of factors and pattern of indicator-factor loadings fit both groups equally well. Both the factor loadings and item thresholds were allowed to be freely estimated in each group. If model fit was adequate, then we proceeded to the next step; otherwise, we stopped. The third step examined loading invariance, which constrained loadings to be equal across both groups. Differences in factor loadings would suggest that items were not assessing the same construct across groups. For example, this test would examine if the items in the Pressure to Eat subscale of the CFQ were associated with comparable relationships to the latent construct (parents pressuring their children to eat) across the gender groups in this sample. If model fit was adequate, then we proceeded to the next step; otherwise, we stopped. The fourth step examined item intercept invariance, which constrained loadings and intercepts to be equal across both groups. Lack of invariance would suggest that the groups had different thresholds for endorsing a particular item, such that one group endorsed the item at higher severity despite having similar levels of the latent construct. For example, Latino parents may produce different raw scores on the items that comprise the Pressure to Eat subscale than non-Latino parents despite having similar global Pressure to Eat subscale scores.

## Results

Means and standard deviations on all subscales across all groups are reported in Table 1.

### Invariance across male and female samples

#### CFQ

Independent CFAs indicated poor fit of the single latent factor for either females or males on Perceived Parent Weight, Restriction, and Pressure to Eat subscales. Throughout gender invariance examination, males were used as the reference group. Results support configural, loading, and intercept invariance for the Concern about Child Weight and Parental Responsibility subscales. This indicates these subscales appear to be assessing the same underlying constructs across males and females, and the groups are endorsing items at similar thresholds. Comparatively, the AIC values indicate that the intercept invariance model appears to be the optimal model for both subscales (Table 2).

**Table 2:** Independent and multi-group CFAs for parent feeding in males and females

	Overall fit indices			Comparative fit
Subscale	χ^2^ (df)	CFI	RMSEA (90% CI)	AIC
Child feeding questionnaire				
Perceived parent weight				
*Female*	*4.063(2), p = .13*	*.960*	*.091 (.000 - .220)*	*–*
*Male*	*37.804(2), p < .01*	*.687*	*.395 (.291 - .509)*	*–*
Concern child weight				
*Female*	*0.000(0)*	*1.000*	*.000*	*–*
*Male*	*0.000(0)*	*1.000*	*.000*	*–*
Configural Invariance	0.000(0)	1.000	.000	36.000
Loading Invariance	1.851(2), *p* = .40	1.000	.000 (.000 - .125)	33.851
Intercept Invariance	4.212(5), *p* = .52	1.000	.000 (.000 - .082)	30.212
Parental responsibility				
*Female*	*0.000(0)*	*1.000*	*.000*	*–*
*Male*	*0.000(0)*	*1.000*	*.000*	*–*
Configural Invariance	0.000(0)	1.000	.000	36.000
Loading Invariance	1.844(2), *p* = .40	1.000	.000 (.000 - .075)	34.756
Intercept Invariance	6.854(5), *p* = .23	.996	.039 (.000 - .104)	32.854
Restriction				
*Female*	*127.385(20), p < .01*	*.711*	*.208 (.174 - .243)*	
*Male*	*200.813(20), p < .01*	*.599*	*.280 (.246 - .316)*	
Pressure to eat				
*Female*	*5.092(2), p = .08*	*.975*	*.112 (.000 - .237)*	
*Male*	*4.140(2), p = .13*	*.981*	*.096 (.000 - .230)*	

*Note.* Italicized analyses represent independent CFAs examining each subscale as a latent single factor. Perceived Parent Weight has 4 indicators, Concern about Child Weight has 3 indicators, Parental Responsibility has 3 indicators, Restriction has 8 indicators and Pressure to Eat has 4 indicators. AIC is a comparative fit index for two or more groups

#### CEBQ

Independent CFAs indicated poor fit of the single latent factor for either females or males on Emotional Overeating, Slowness in Eating, Emotional Undereating and Food Fussiness. Results support configural, loading, and intercept invariance for the Food Responsiveness, Enjoyment of Food, and Satiety Responsiveness subscales indicating these subscales appear to be assessing the same underlying constructs across males and females. In addition, males and females appear to be endorsing items at similar thresholds. The AIC values indicated the intercept invariance model to be the optimal model for all of these subscales. However, Desire to Drink only achieved configural and loading, with the model poorly fitting for intercept invariance. The AIC value further confirms the configural invariance model to be the optimal model for Desire to Drink Table 3.

**Table 3:** Independent and multi-group CFAs for child eating in males and females

	Overall fit indices			Comparative fit
Subscale	χ^2^ (df)	CFI	RMSEA (90% CI)	AIC
Child eating behaviour questionnaire				
Food responsiveness				
*Female*	*6.485(5), p = .26*	*.991*	*.049 (.000 - .141)*	*–*
*Male*	*6.844(5), p = .23*	*.991*	*.057 (.000 - .150)*	*–*
Configural Invariance	13.329(10), *p* = .21	.991	.037 (.000-.084)	73.329
Loading Invariance	14.308(14), *p* = .43	.999	.010 (.000-.064)	66.308
Intercept Invariance	14.661(19), *p* = .74	1.000	.000 (.000-.041)	56.661
Emotional overeating				
*Female*	*2.280(2), p = .32*	*.999*	*.034 (.000 - .185)*	*–*
*Male*	*8.973(2), p = .01*	*.977*	*.174 (.070 - .297)*	*–*
Enjoyment of food				
*Female*	*0.462(2), p = .79*	*1.000*	*.000 (.000 - .113)*	*–*
*Male*	*1.041(2), p = .59*	*1.000*	*.000 (.000 - .153)*	*–*
Configural Invariance	1.504(4), *p* = .83	1.000	.000 (.000 - .058)	49.504
Loading Invariance	3.590(7), *p* = .83	1.000	.000 (.000 - .048)	45.590
Intercept Invariance	3.988(11), *p* = .97	1.000	.000 (.000 - .000)	37.988
Desire to drink				
*Female*	*0.000(0)*	*1.000*	*.000*	*–*
*Male*	*0.000(0)*	*1.000*	*.000*	*–*
Configural Invariance	0.000(0)	1.000	.000	36.000
Loading Invariance	6.196(2), *p* = .05	.991	.094 (.012 - .182)	38.196
Intercept Invariance	14.395(5), *p* = .01	.979	.089 (.037 - .144)	40.395
Satiety responsiveness				
*Female*	*2.429(5), p = .79*	*1.000*	*.000 (.000 - .082)*	*–*
*Male*	*2.271(5), p = .81*	*1.000*	*.000 (.000 - .080)*	*–*
Configural Invariance	4.700(10), *p* = .91	1.000	.000 (.000 - .028)	64.700
Loading Invariance	9.674(14), *p* = .79	1.000	.000 (.000 - .042)	61.674
Intercept Invariance	12.222(19), *p* = .88	1.000	.000 (.000 - .029)	54.222
Slowness in eating				
*Female*	*6.864(2), p = .03*	*.932*	*.140 (.035 - .261)*	*–*
*Male*	*18.374(2), p < .01*	*.909*	*.267 (.164 - .384)*	*–*
Emotional undereating				
*Female*	*6.013(2), p = .05*	*.961*	*.127 (.005 - .250)*	*–*
*Males*	*6.481(2), p = .04*	*.967*	*.140 (.027 - .266)*	*–*
Food fussiness				
*Female*	*55.537(9), p < .01*	*.880*	*.204 (.155 - .257)*	*–*
*Male*	*35.779(9), p < .01*	*.932*	*.161 (.108 - .218)*	*–*

*Note.* Italicized analyses represent independent CFAs examining each subscale as a single latent factor. Food Responsiveness has 5 indicators, Emotional Overeating has 4 indicators, Enjoyment of Food has 4 indicators, Desire to Drink has 3 indicators, Satiety Responsiveness has 5 indicators, Slowness in Eating has 4 indicators, Emotional Undereating has 4 indicators, and Food Fussiness has 6 indicators. AIC is a comparative fit index for two or more groups

### Invariance across Latino and non-Latino samples

#### CFQ

Independent CFAs indicated poor fit of the single latent factor for either Latinos or Non-Latinos on Perceived Parent Weight, Restriction, and Pressure to Eat subscales. Results support configural, loading, and intercept invariance for Concern about Child Weight subscale. This subscale appears to be assessing the same underlying construct across ethnic groups, and the groups appear to be endorsing items at similar thresholds. Comparatively, the loading invariance model appears to be the optimal model for this subscale. Parental Responsibility achieved configural invariance but not loading invariance (Table 4).

**Table 4:** Independent and multi-group CFAs for parent feeding in non-Latino and Latino samples

	Overall fit indices			Comparative fit
Subscale	χ^2^ (df)	CFI	RMSEA (90% CI)	AIC
Child feeding questionnaire				
Perceived parent weight				
*Non-Latino*	*38.046(2), p < .01*	*.734*	*.337 (.248 - .434)*	*–*
*Latino*	*2.509(2), p = .29*	*.986*	*.056 (.000 - .237)*	*–*
Concern child weight				
*Non-Latino*	*0.000(0)*	*1.000*	*.000*	*–*
*Latino*	*0.000(0)*	*1.000*	*.000*	*–*
Configural Invariance	0.000(0)	1.000	.000	36.000
Loading Invariance	0.152(2), *p* = .93	1.000	.000 (.000 - .041)	32.152
Intercept Invariance	9.751(5), *p* = .08	.984	.063 (.000 - .122)	35.751
Parental responsibility				
*Non-Latino*	*0.000(0)*	*1.000*	*.000*	*–*
*Latino*	*0.000(0)*	*1.000*	*.000*	–
Configural Invariance	0.000(0)	1.000	.000	36.000
Loading Invariance	10.143(2), *p* < .01	.981	.131 (.059 - .215)	42.143
Restriction				
*Non-Latino*	*221.360(20), p < .01*	*.547*	*.252 (.222 - .282)*	*–*
*Latino*	*147.769(20), p < .01*	*.680*	*.283 (.241 - .326)*	*–*
Pressure to eat				
*Non-Latino*	*3.787(2), p = .15*	*.990*	*.075 (.000 - .190)*	*–*
*Latino*	*5.446(2), p = .07*	*.939*	*.147 (.000 - .301)*	*–*

*Note.* Italicized analyses represent independent CFAs examining each subscale as a single factor. Perceived Parent Weight has 4 indicators, Concern about Child Weight has 3 indicators, Parental Responsibility has 3 indicators, Restriction has 8 indicators and Pressure to Eat has 4 indicators. AIC is a comparative fit index for two or more groups

#### CEBQ

Independent CFAs indicated poor fit of the single latent factor for either Latinos or Non-Latinos on Slowness in Eating, Emotional Undereating, and Food Fussiness. Results support configural, loading, and intercept invariance for all other scales. For Food Responsiveness, the best fitting model was the loading invariance model when compared to the other models. For Emotional Overeating, the configural invariance model was the best fitting of the three invariance models. For Enjoyment of food, Desire to Drink, and Satiety Responsiveness the intercept model was the optimal model with the lowest AIC values (Table 5).

**Table 5:** Independent and multi-group CFAs for child eating in Non-Latino and Latinos

	Overall fit indices			Comparative fit
Subscale	χ^2^ (df)	CFI	RMSEA (90% CI)	AIC
Child eating behaviour questionnaire				
Food responsiveness				
*Non-Latino*	*6.408(5), p = .27*	*.992*	*.042 (.000 - .124)*	*–*
*Latino*	*4.478(5), p = .48*	*1.000*	*.000 (.000 - .147)*	*–*
Configural Invariance	10.892(10), *p* = .37	.998	.019 (.000 - .074)	70.892
Loading Invariance	12.700(14), *p* = .55	1.000	.000 (.000 - .057)	64.700
Intercept Invariance	28.388(19), *p* = .08	.974	.045 (.000 - .078)	70.388
Emotional overeating				
*Non-Latino*	*2.467(2), p = .29*	*.999*	*.038 (.000 -.167)*	*–*
*Latino*	*0.339(2), p = .84*	*1.000*	*.000 (.000 -.124)*	*–*
Configural Invariance	2.803(4), *p* = .59	1.000	.000 (.000 -.083)	50.803
Loading Invariance	12.486(7), *p* = .09	.990	.057 (.000 - .108)	54.486
Intercept Invariance	22.522(11), *p* = .02	.980	.066 (.025 - .105)	56.522
Enjoyment of food				
*Non-Latino*	*1.600(2), p = .45*	*1.000*	*.000 (.000 - .147)*	*–*
*Latino*	*0.539(2), p = .76*	*1.000*	*.000 (.000 - .149)*	*–*
Configural Invariance	2.139(4), *p* = .71	1.000	.000 (.000 -.072)	50.139
Loading Invariance	4.800(7), *p* = .68	1.000	.000 (.000 - .062)	46.800
Intercept Invariance	9.141(11), *p* = .61	1.000	.000 (.000 - .059)	43.141
Desire to drink				
*Non-Latino*	*0.000(0)*	*1.000*	*.000*	*–*
*Latino*	*0.000(0)*	*1.000*	*.000*	*–*
Configural Invariance	0.000(0)	1.000	.000	36.000
Loading Invariance	0.344(2), *p* = .84	1.000	.000 (.000 - .072)	32.344
Intercept Invariance	3.740(5), *p* = .59	1.000	.000 (.000 - .077)	29.740
Satiety responsiveness				
*Non-Latino*	*1.689(5), p = .89*	*1.000*	*.000 (.000 - .050)*	*–*
*Latino*	*4.471(5), p = .45*	*1.000*	*.000 (.000 - .151)*	*–*
Configural Invariance	6.420(10), *p* = .78	1.000	.000 (.000 - .048)	66.420
Loading Invariance	13.890(14), *p* = 46	1.000	.000 (.000 - .062)	65.890
Intercept Invariance	22.116(19), *p* = .28	.988	.026 (.000 - .065)	64.116
Slowness in eating				
*Non-Latino*	*21.501(2), p < .01*	*.861*	*.248 (.160 - .347)*	*–*
*Latino*	*3.795(2), p = .15*	*.980*	*.106 (.000 -.268)*	*–*
Emotional undereating				
*Non-Latino*	*11.464(2), p < .01*	*.951*	*.173 (.085 - .275)*	*–*
*Latino*	*2.221(2), p = .33*	*.995*	*.037 (.000 - .228)*	*–*
Food fussiness				
*Non-Latino*	*57.979(9), p < .01*	*.916*	*.185 (.141 - .232)*	*–*
*Latino*	*22.373(9), p < .01*	*.928*	*.136 (.066 - .208)*	*–*

*Note.* Italicized analyses represent independent CFAs examining each subscale as a single latent factor. Food Responsiveness has 5 indicators, Emotional Overeating has 4 indicators, Enjoyment of Food has 4 indicators, Desire to Drink has 3 indicators, Satiety Responsiveness has 5 indicators, Slowness in Eating has 4 indicators, Emotional Undereating has 4 indicators, and Food Fussiness has 6 indicators. AIC is a comparative fit index for two or more groups

### Invariance across food secure and insecure households

#### CFQ

Independent CFAs indicated poor fit of the single latent factor for either food secure or insecure households on Perceived Parent Weight, Restriction, and Pressure to Eat. Results support configural, loading and intercept invariance for Concern about Child Weight, with the intercept invariance model being the best fitting based on AIC values. For the Parental Responsibility subscale, only configural invariance was achieved, with loading invariance model fitting poorly (Table 6).

**Table 6:** Independent and multi-group CFAs for parent feeding in food secure and insecure households

	Overall fit indices			Comparative fit
Subscale	χ^2^ (df)	CFI	RMSEA (90% CI)	AIC
Child feeding questionnaire				
Perceived parent weight				
*Food Secure*	*36.347(2), p < .01*	*.724*	*.322 (.235 - .417)*	*–*
*Food Insecure*	*2.912(2), p = .23*	*.973*	*.080 (.000 - .263)*	*–*
Concern child weight				
*Food Secure*	*0.000(0)*	*1.000*	*.000*	*–*
*Food Insecure*	*0.000(0)*	*1.000*	*.000*	*–*
Configural Invariance	0.000(0)	1.000	.000	36.000
Loading Invariance	3.195(2), *p* = .20	.996	.050 (.000 - .148)	35.195
Intercept Invariance	6.362(5), *p* = .27	.996	.034 (.000 - .101)	32.362
Parental responsibility				
*Food Secure*	*0.000(0)*	*1.000*	*.000*	*–*
*Food Insecure*	*0.000(0)*	*1.000*	*.000*	*–*
Configural Invariance	0.000)0)	1.000	.000	36.000
Loading Invariance	8.605(2), *p* = .01	.986	.118 (.046 - .204)	40.605
Restriction				
*Food Secure*	*207.007 (20), p < .01*	*.651*	*.237 (.209 - .267)*	*–*
*Food Insecure*	*92.144 (20), p < .01*	*.706*	*.225 (.180 - .273)*	*–*
Pressure to eat				
*Food Secure*	*1.848(2), p = .40*	*1.000*	*.000 (.000 - .150)*	*–*
*Food Insecure*	*9.570(2), p < .01*	*.911*	*.231 (.099 - .386)*	*–*

*Note.* Italicized analyses represent independent CFAs examining each subscale as a single factor. Perceived Parent Weight has 4 indicators, Concern about Child Weight has 3 indicators, Parental Responsibility has 3 indicators, Restriction has 8 indicators and Pressure to Eat has 4 indicators. AIC is a comparative fit index for two or more groups

#### CEBQ

Independent CFAs indicated poor fit of the single latent factor for either food secure or insecure households on Food Responsiveness, Emotional Overeating, Slowness in Eating, Emotional Undereating, and Food Fussiness. The models for Enjoyment of Food, Desire to Drink, and Satiety Responsiveness all supported the configural, loading and intercept invariance. Thus, these subscales appear to be assessing the same underlying constructs and samples are endorsing items at similar threshold levels. Based on the AIC index, the intercept invariance model for Enjoyment of Food, the loading invariance model for Desire to Drink, and configural invariance model for Satiety Responsiveness are the optimal models (Table 7).

**Table 7:** Independent and multi-group CFAs for child eating in food secure and insecure households

	Overall fit indices			Comparative fit
Subscale	χ^2^ (df)	CFI	RMSEA (90% CI)	AIC
Child eating behaviour questionnaire				
Food responsiveness				
*Food Secure*	*7.902(5), p = .16*	*.985*	*.059 (.000 - .133)*	*–*
*Food Insecure*	*14.460(5), p* = .01	.939	.163 (.068 - .265)	–
Emotional overeating				
*Food Secure*	*9.252(2), p = .01*	*.982*	*.148 (.062 - .250)*	*–*
*Food Insecure*	*3.071(2), p = .22*	*.994*	*.087 (.000 - .267)*	*–*
Enjoyment of food				
*Food Secure*	*0.919(2), p = .63*	*1.000*	*.000 (.000 - .122)*	*–*
*Food Insecure*	*1.103(2), p = .58*	*1.000*	*.000 (.000 - .198)*	*–*
Configural Invariance	2.026(4), *p* = .73	1.000	.000 (.000 - .071)	50.026
Loading Invariance	2.618(7), *p* = .92	1.000	.000 (.000 - .029)	44.618
Intercept Invariance	5.375(11), *p* = .91	1.000	.000 (.000 - .026)	39.375
Desire to drink				
*Food Secure*	*0.000(0)*	*1.000*	*.000*	*–*
*Food Insecure*	*0.000(0)*	*1.000*	*.000*	*–*
Configural Invariance	0.000(0)	1.000	.000	36.000
Loading Invariance	0.038(2), *p* = .98	1.000	.000 (.000 - .000)	32.038
Intercept Invariance	11.416(5), *p* = .04	.985	.074 (.012 - .131)	37.416
Satiety responsiveness				
*Food Secure*	*2.167(5), p = .83*	*1.000*	*.000 (.000 - .064)*	*–*
*Food Insecure*	*4.392(5), p = .49*	*1.000*	*.000 (.000 - .154)*	*–*
Configural Invariance	6.579(10), *p = .77*	*1.000*	*.000 (.000 - .049)*	*66.579*
Loading Invariance	20.049(14), *p* = .13	.976	.043 (.000 - .082)	72.049
Intercept Invariance	25.077(19), *p* = .16	.976	.037 (.000 - .072)	67.077
Slowness in eating				
*Food Secure*	*17.342(2), p < .01*	*.904*	*.215 (.129 - .313)*	*–*
*Food Insecure*	*2.063(2), p = .36*	*.999*	*.021 (.000 - .237)*	*–*
Emotional undereating				
*Food Secure*	*2.852(2), p = .24*	*.996*	*.051 (.000 - .171)*	*–*
*Food Insecure*	*10.301(2), p < .01*	*.843*	*.242 (.111 - .397)*	*–*
Food fussiness				
*Food Secure*	*27.170(9), p < .01*	*.969*	*.110 (.064 - .159)*	*–*
*Food Insecure*	*48.759(9), p < .01*	*.797*	*.249 (.183 - .320)*	*–*

*Note.* Italicized analyses represent independent CFAs examining each subscale as a single latent factor. Food Responsiveness has 5 indicators, Emotional Overeating has 4 indicators, Enjoyment of Food has 4 indicators, Desire to Drink has 3 indicators, Satiety Responsiveness has 5 indicators, Slowness in Eating has 4 indicators, Emotional Undereating has 4 indicators, and Food Fussiness has 6 indicators. AIC is a comparative fit index for two or more groups

## Discussion

Although there have been extensive studies that make group comparisons on child eating and feeding practices, few studies have examined measures to ensure group comparisons are equivalent across important group characteristics related to childhood obesity. Of note, there have been association studies relating minority groups’ responses and scores on child eating and feeding practices measures to childhood obesity with limited research examining the appropriateness of these measures among minority groups. To further strengthen the research base for assessing child feeding practices and eating behaviors, we sought to evaluate the factor structure and measurement invariance of the CFQ and CEBQ across gender and ethnicity. A unique contribution of our study was the examination of household food security. It is important to ensure that child eating and feeding practices measures perform consistently across diverse environments.

Overall, results regarding the factor structure yielded mixed results for each measure and highlight some important issues to consider in assessing child eating and feeding practices. For the CFQ, the factor structures did not differ across any of the groups for the subscales Concern about Child Weight and Parental Responsibility. Our study is consistent with and adds to the existing psychometric literature. Cumulatively, Concern about Child Weight and Parent Responsibility, are invariant across Latinos (current study, [14]), African Americans [14], preschool-aged boys and girls (current study, [14, 27]) and diverse food secure environments [current study]. Most notably, the factor structures for Restriction, and Pressure to Eat from the CFQ varied across the ethnic and food security groups in the current study. It is important to note our findings add to the existing literature. There have been cross-cultural conceptual issues for the Restriction subscale among samples of Latinos (current study, [14]), African American [14, 40] and an Australian sample [27], and diverse food secure groups [current study]. It is important to note, that all of these studies, including the current study, assessed parents of preschool age children. For Pressure to Eat subscale, cross-cultural issues were found in the current study and Boles and colleagues [44], but Anderson and colleagues [14] found this scale to be invariant. In our study, the Perceived Parent Weight factor fit poorly across all the groups. Consistent with our findings, Anderson et al. [14] found issues with this factor and subsequently dropped it altogether. Given all of these findings, caution should be given to conclusions derived from studies that used these subscales across food secure samples [17, 18]. Similarly, the lack of measurement invariance on Pressure to Eat and Restriction suggests findings from published studies demonstrating higher rates among Latinos compared to other ethnic and racial groups (i.e., [11, 16]) should be interpreted with caution. Overall, results of the current study suggest that research should continue to validate the CFQ.

Results on the CEBQ revealed that three of the eight factors (Enjoyment of Food, Desire to Drink, and Satiety Responsiveness) performed well and did not vary across any of the groups. However, the intercepts did vary for Food Responsiveness where Latinos report lower thresholds for endorsing these items. In contrast, the intercepts varied for Emotional Overeating as well, but Latinos reported higher thresholds for endorsing these items when compared to non-Latino Whites. Importantly, the Food Fussiness factor showed poor fit across all of the groups. Other factors (i.e., Desire to Drink, Slowness in Eating, and Emotional Undereating) varied across the groups, suggesting the items in these subscales do not assess the same underlying construct across groups.

Cumulatively, the findings from the psychometric research on the CEBQ are mixed. Domoff et al. [22] conducted a validation study of the CEBQ among an ethnically diverse sample of low-income parents of preschool age children within the United States and replicated the original factor structure. A second study conducted on predominantly Hispanic and Black parents of preschool age children within the United States failed to replicate the original factor structure of the CEBQ and proposed three new factors [23]. Consistent with Domoff et al. [22], our study found that three of the food approach subscales (Food Responsiveness, Emotional Overeating, Enjoyment of Food) and one food avoidance subscale (Satiety Responsiveness) performed well across gender and ethnicity. But, consistent with Sparks & Radnitz [23], Food Fussiness, Slowness in Eating, and Emotional Undereating had cross-cultural conceptual problems. It is important to note that all studies used parents of preschool age children, but differed in mode of administration. The current study along with Sparks & Radnitz [23] administered self-report questionnaires while Domoff and colleagues [22] administered the questionnaire orally. At best, the collective research within the United States is inconclusive with regards to the construct validity of the CEBQ and highlights the need for further research.

A unique contribution of this study was an examination of measurement invariance across food secure and insecure households. For the CEBQ, Food responsiveness, which assesses external eating (e.g., responsiveness to sight, smell, and taste of palatable foods), and Food Fussiness, which assesses picky eating, failed to fit the data adequately. A closer examination of participants from food insecure households and their responses to food responsiveness and food fussiness items revealed higher rates of “untrue” endorsements. In addition, some parents wrote comments on these items stating those behaviors or situations did not occur. This might suggest that within food insecure environments, items from these subscales do not apply or fail to capture the living context of these families. In other words, the behaviors assessed in these items might be specific to households with consistent and stable food availability. Similarly, parental food restriction also may be only applicable in food secure environments given that within food insecure households the family economics and resources are placing food restrictions on the family. It is important to highlight that household food security is associated with income, however, food insecurity in children can still be quite high at incomes that are two or three times the poverty level [2]. Similarly, caregiver disability can influence the risk of food insecurity in children, but high rates can still be evident among households with employed caregivers [2]. Recent research has found that numerous factors aside from income and employment can influence household food insecurity including caregiver incarceration, immigration status, and caregiver’s mental and physical health ([2] for a review). Since these analyses are unique to our study, replication is needed. Further research is need to explore how the presence of poor caregiver mental health or disability influences parent-report of child eating behaviors, if at all.

The results of this study should be interpreted in light of several limitations. First, the findings are limited to White and Latino samples fluent in English who are parents of preschool aged children. Second, the data are parent-report and may be influenced by context-specific eating behaviors and/or the desire to respond with socially expected answers. The very low food insecure and low food insecure were combined into one food insecure group due to sample size. Future research should consider separating this group as the very low food insecure group may differ significantly in feeding practices than the other groups. A larger sample size would have allowed for examining the entire factor structure of the scales within one CFA analysis. Relatedly, the number of analyses conducted increase the chance of family-wise error rate and the probability of making a type I error. This signifies that some of the *p*-values were significant simply by chance. Furthermore, this study was cross-sectional in nature and cannot address longitudinal measurement invariance or distinguish between important differences among individuals that exist within specific racial groups, genders, or food security groups (i.e., genetics or individual experiences). Future research should consider conducting longitudinal measurement invariance with these variables, as this can address if changes on measurement over time reflect individual change or change in the properties of the measurement instrument.

## Conclusions

In summary, continued psychometric research and scale refinement is needed on the CFQ and CEBQ. For the CFQ, Concern about Child Weight and Parental Responsibility subscales perform consistently across gender, ethnicity, and food secure environments. For the CEBQ, Enjoyment of Food, Desire to Drink, and Satiety Responsiveness subscales were invariant across all the contextual factors. Given that pediatric obesity is influenced by contextual factors such as gender, food security, and ethnicity, it is imperative that assessments perform consistently across these factors. The ability to assess risk factors for pediatric obesity, or to detect change across time in treatment studies or longitudinal studies, is compromised if the measures used are influenced by contextual factors.

## Abbreviations

BMI: Body mass index
CEBQ: Children’s Eating Behaviour Questionnaire
CFA: Confirmatory factor analysis
CFI: Comparative fit index
CFQ: Child Feeding Questionnaire
RMSEA: Root mean square error of approximation
